# Network analysis of the depressive symptom profiles in Asian patients with depressive disorders: Findings from the Research on Asian Psychotropic Prescription Patterns for Antidepressants (REAP‐AD)

**DOI:** 10.1111/pcn.12989

**Published:** 2020-03-05

**Authors:** Seon‐Cheol Park, Eun Young Jang, Yu‐Tao Xiang, Shigenobu Kanba, Takahiro A. Kato, Mian‐Yoon Chong, Shih‐Ku Lin, Shu‐Yu Yang, Ajit Avasthi, Sandeep Grover, Roy A. Kallivayalil, Pichet Udomratn, Kok Yoon Chee, Andi J. Tanra, Chay‐Hoon Tan, Kang Sim, Norman Sartorius, Yong Chon Park, Naotaka Shinfuku

**Affiliations:** ^1^ Department of Psychiatry Inje University Haeundae Paik Hospital Busan Republic of Korea; ^2^ Department of Counseling Psychology Honam University College of Humanities and Social Sciences Gwangju Republic of Korea; ^3^ Faculty of Health Sciences University of Macau Macau China; ^4^ Department of Neuropsychiatry, Graduate School of Medicine Kyushu University Fukuoka Japan; ^5^ Department of Psychiatry, Kaohsiung Chang Gung Memorial Hospital Kaohsiung & Chang Gung University School of Medicine Linkou Taiwan; ^6^ Psychiatry Center Tapei City Hospital Taipei Taiwan; ^7^ Department of Pharmacy Taipei City Hospital and Fu Jen University Taipei Taiwan; ^8^ Department of Psychiatry Post Graduate Institute of Medical Education and Research (PGIMER) Chandigarh India; ^9^ Pushpagiri Institute of Medical Sciences Tiruvalla India; ^10^ Department of Psychiatry, Faculty of Medicine Prince of Songkla University Songkhla Thailand; ^11^ Tunku Abdul Rahman Institute of Neurosciences Kuala Lumpur Malaysia; ^12^ Faculty of Medicine, Department of Psychiatry Hasanuddin University Makassar Indonesia; ^13^ Department of Pharmacology National University Hospital Singapore; ^14^ Institute of Mental Health Buangkok Green Medical Park Singapore; ^15^ Association for the Improvement of Mental Health Programmes Geneva Switzerland; ^16^ Department of Neuropsychiatry Hanyang University Guri Hospital Guri Republic of Korea; ^17^ Department of Social Welfare, School of Human Sciences Seinan Gakuin University Fukuoka Japan

**Keywords:** Asian, depressive disorders, depressive symptoms, network analysis, node strength centrality

## Abstract

**Aim:**

We aimed to estimate the network structures of depressive symptoms using network analysis and evaluated the geographic regional differences in theses network structures among Asian patients with depressive disorders.

**Methods:**

Using data from the Research on Asian Psychotropic Prescription Patterns for Antidepressants (REAP‐AD), the network of the ICD‐10 diagnostic criteria for depressive episode was estimated from 1174 Asian patients with depressive disorders. The node strength centrality of all ICD‐10 diagnostic criteria for a depressive episode was estimated using a community‐detection algorithm. In addition, networks of depressive symptoms were estimated separately among East Asian patients and South or Southeast Asian patients. Moreover, networks were estimated separately among Asian patients from high‐income countries and those from middle‐income countries.

**Results:**

Persistent sadness, fatigue, and loss of interest were the most centrally situated within the network of depressive symptoms in Asian patients with depressive disorders overall. A community‐detection algorithm estimated that when excluding psychomotor disturbance as an outlier, the other nine symptoms formed the largest clinically meaningful cluster. Geographic and economic variations in networks of depressive symptoms were evaluated.

**Conclusion:**

Our findings demonstrated that the typical symptoms of the ICD‐10 diagnostic criteria for depressive episode are the most centrally situated within the network of depressive symptoms. Furthermore, our findings suggested that cultural influences related to geographic and economic distributions of participants could influence the estimated depressive symptom network in Asian patients with depressive disorders.

The heterogeneity of depressive syndrome can be due to the polythetic and operational definition of ‘depressive syndrome’ from the viewpoint of a categorical approach rather than a dimensional approach.^1^, ^2^ Thus, the heterogeneity of depressive syndrome can be criticized in terms of Wittgenstein's ‘game’ analogy as follows: Whereas cases of depressive syndrome are not commonly underpinned with an ‘essence,’ they are connected by the ‘family resemblance,’ which denotes extensions of meaning.^3^ Thus, although a simpler definition of ‘major depressive disorder’ (MDD) that eliminates the four somatic symptoms from its diagnostic criteria in the DSM‐IV^4^ has been proposed,^5^, ^6^, ^7^ the definition was not further simplified during the DSM‐5 revision process.

As shown in Table 1, according to the ICD‐10,^8^ the operational diagnostic criteria for depressive episode consists of (i) the typical symptoms, including depressed symptoms, loss of interest, and reduced energy; and (ii) the other common symptoms, including reduced concentration and attention, reduced self‐esteem and self‐confidence, ideas of guilt and unworthiness, bleak and pessimistic views of the future, ideas or acts of self‐harm or suicide, disturbed sleep, and disturbed appetite. The severity of depressive episodes varies with the number and severity of depressive symptoms. According to the DSM‐5,^9^ the operational diagnosis of MDD consists of: (i) the presence of at least one of the core symptoms of depressed mood and loss of interest or pleasure; and (ii) the presence of five or more of the core symptoms and the other depressive symptoms, including weight loss or gain, insomnia or hypersomnia, psychomotor agitation or retardation, fatigue or loss of energy, feelings of worthlessness or excessive guilt, diminished ability to concentrate or indecisiveness, and recurrent thoughts of death or recurrent suicidal ideation. As shown in Table 1, the main differences in the ICD‐10 criteria for depressive episodes and DSM‐5 criteria for MDD are as follows: First, the DSM‐5 criteria for depressive mood cover the two symptoms of depressive mood and bleak and pessimistic views for the future included in the ICD‐10 criteria. Hopelessness, which can be partly consistent with bleak and pessimistic views in the ICD‐10 criteria, has been newly added as a subjective descriptor to depressive moods in the revision from DSM‐IV to DSM‐5.^10^ Second, in the ICD‐10 criteria, low self‐esteem, low self‐reproach, suicidality, and vegetative symptoms are regarded as better indicators for severity than other symptoms, whereas all symptoms are equally treated in the DSM‐5 criteria.^11^

**Table 1:** DSM‐5 criteria for major depressive disorder vs ICD‐10 criteria for depressive episodes

DSM‐5	ICD‐10
Depressed mood† Markedly diminished interest or pleasure† Significant weight loss or weight gain Insomnia or hypersomnia Psychomotor agitation or retardation Fatigue or loss of energy Feelings of worthlessness or excessive or inappropriate guilt Diminished ability to think or concentrate, or indecisiveness Recurrent thoughts of death, recurrent suicidal ideation, or a suicide attempt	Depressed mood‡ Loss of interest and enjoyment‡ Reduced energy and diminished activity‡ Reduced concentration and attention Reduced self‐esteem and self‐confidence Ideas of guilt and unworthiness Bleak and pessimistic views of the future Ideas or acts of self‐harm or suicide Disturbed sleep Diminished appetite

† Core symptoms of the DSM‐5 criteria for major depressive disorder.

‡ Core symptoms of the ICD‐10 criteria for depressive episode.

In terms of the heterogeneity of the depressive syndrome, 227 different symptom combinations fulfilling the DSM‐5 diagnostic criteria for MDD can be theoretically calculated.^12^, ^13^ However, in actual clinical practice, among 1566 patients with MDD in the Rhode Island Methods to Improve Diagnostic Assessment and Services (MIDAS) project^13^ and 853 patients with MDD in the Clinical Research Center for Depression (CRESCEND) study,^14^ only 170 and only 119 different symptom combinations were identified, respectively. It is thus plausible that interrelated symptom constellations can be established within the psychopathology of depressive syndrome.

The network approach has recently been suggested as a computational method to explain the complexity of psychiatric disorders, for the following reasons^15^, ^16^: First, the pathways between variables can be explored and novel and interesting relationships can be identified by visualization methods used in network psychiatry. Second, the properties of the network as a whole can be evaluated using the network approach. Third, the variables that are disproportionally related with the network's adaptive functioning can be identified by network psychiatry. Thus, the network approach has been used to reveal a collection of interrelated symptoms within an entire network.^17^, ^18^ Moreover, the network analysis approach focuses on the network of relationships among symptoms but not the observation for manifestations of an underlying disease.^17^ While the standard reductionist model is based on the typical top‐down process that ‘symptoms are the constituent factors of an underlying disease,’ the network analysis approach basically assumes the bottom‐up process that ‘symptoms and associations among them are the disease itself.’^19^, ^20^ Therefore, while the structural equation model states that the common influence of a latent variable can explain the covariance of constituent symptoms, the network analysis approach states that a network of symptom components is regarded as a psychiatric constitute.^21^, ^22^, ^23^ From the viewpoint of the Wittgensteinian analogy of the language game, cases of the depressive syndrome are connected by the extension of meaning but not underlying essence. Existence of the relevance of the mental process for a distinctive diagnostic entity may be denied in terms of the heterogeneity of the depressive syndrome.^3^ Hence, unlike the structural equation model, the network analysis approach can present a novel aspect of the intertwined and interrelated symptoms within the network of depressive symptoms consistent with nominalism but not essentionalism.^24^, ^25^ From the perspective of a network approach, it is speculated that the central symptoms may be more influential than peripheral symptoms and facilitate the interrelated symptoms within the entire network.^26^, ^27^, ^28^ Fried *et al*.^29^ reported that the DSM symptoms are not more central than non‐DSM symptoms within a network of depressive symptoms, based on a study of 3462 outpatients with depressive disorders in the Sequenced Treatment Alternatives to Relieve Depression (STAR*D) study. In addition, the findings of Fried *et al*.^29^ have been replicated among 5952 Han Chinese women who fulfilled the DSM‐IV criteria for MDD.^30^

A strong connection of depressive symptoms or emotions has been suggested as an appropriate method for exploring the organization of symptomatology in depressive disorders.^31^, ^32^ Moreover, it has been suggested that the patho‐facilitative or patho‐reactive influences of specific cultures can result in international differences in clinical manifestations of depressive disorders.^33^ Hence, using data from the Research on Asian Psychotropic Prescription Patterns for Antidepressants (REAP‐AD) survey, which is one of the largest international research collaborations within Asia,^34^, ^35^, ^36^, ^37^ we aimed to estimate the network structures of depressive symptoms and evaluate the geographic regional differences in these network structures among Asian patients with depressive disorders.

## Methods

### Study overviews and participants

As described elsewhere,^34^, ^35^, ^36^, ^37^ in the REAP‐AD survey, 2470 psychiatric patients who had been treated with antidepressants were recruited from 10 Asian countries or special administrative regions – China, Hong Kong, India, Indonesia, Japan, Korea, Malaysia, Singapore, Taiwan, and Thailand – during the survey period from March to June 2013. Antidepressants were defined as psychoanaleptics, which were coded as N06A in the Anatomical and Therapeutic Chemical (ATC) classification system. The group comprising preparations used in the treatment of endogenous and exogenous depression was denoted as N06A antidepressants.^38^ A consensus meeting was held to ensure the consistency of evaluating the clinical characteristics of the study participants before the initiation of the REAP‐AD study. To estimate the network structures of data from the REAP‐AD in this study, we selected those participants who fulfilled the following criteria: (i) diagnosis of a depressive episode (F32) or recurrent depressive disorder (F33) according to the ICD‐10^8^ by the psychiatrists; and (ii) availability of the presence or absence of the 10 depressive symptom profiles defined by the ICD‐10 diagnostic criteria for depression.^8^ Consequently 1174 Asian patients with depressive episode or recurrent depressive disorder were included for network analyses of depressive symptoms.

### Depressive symptom profiles and geographic and economic classifications of countries

Based on the ICD‐10 diagnostic criteria for depression,^8^ each of the 10 depressive symptoms was evaluated as present or absent within each participant. The depressive symptom profiles listed in the National Institute for Health and Care Excellence (NICE) guidelines for depression were persistent sadness or low mood (SAD), loss of interest or pleasure (INT), fatigue or low energy (FAT), disturbed sleep (SLE), poor concentration or indecisiveness (CON), low self‐confidence (SEL), decreased or increased appetite (APE), suicidal thoughts or acts (SUI), agitated or slowed movements (AGI), and guilt or self‐blame (GUI).^39^ According to the ICD‐10 diagnostic criteria for depression,^8^ persistent sadness or low mood, loss of interest or pleasure, and fatigue or low energy were regarded as the most typical symptoms of depressive disorders.

According to the United Nations (UN) classification, 10 countries or special administrative regions (SAR) were geographically classified into East Asia (China, Hong Kong, Japan, Korea, and Taiwan) and South or Southeast Asia (India, Indonesia, Malaysia, Singapore, and Thailand). Using the World Bank income designation, countries or SAR were also economically classified into high‐ (Hong Kong, Japan, Korea, Singapore, and Taiwan) and middle‐income countries (China, India, Indonesia, Malaysia, and Thailand).

### Statistical analysis

Using the R‐package qgraph,^40^ the network structures of the 10 depressive symptom profiles listed in the NICE guidelines for depression were estimated. All the depressive symptoms were considered to be dichotomized‐categorical data. Network analyses were performed using polychoric correlations. Depressive symptom network structures, which consisted of both nodes (symptoms) and edges (associations among symptoms), were estimated in Asians overall and East Asians and South or Southeast Asians separately. Furthermore, the network structures were estimated in Asian patients from high‐income countries and those from middle‐income countries separately. False positive edges were controlled using the least absolute shrinkage and selection operator (LASSO).^41^ Thus, the very small edges were set exactly to zero. Using the graphical LASSO (GLASSO) procedures in a network in which the edges were partial correlation coefficients, the average edge was defined as the relationship level between two symptoms, while controlling for all other relationships within the network. Using shrinkage parameters, the extended Bayesian information criterion was minimized and the underlying network structures were recovered.^42^, ^43^ Using the Fruchterman–Reingold algorithm, the stronger connected nodes were placed closer together and the network was represented graphically. Using a modularity‐based community‐detecting algorithm, we investigated whether nodes clustered together. Using the spin‐glass algorithm, we tested whether the number and weighted strength of edges within a cluster exceeded those within another cluster in terms of the communities in the network.^44^ Additionally, the spin‐glass community function of the R‐package igrah was applied over the GLASSO network (weights = null, vertex = null, parupdate = false, gamma = 0.5, start temperature = 1, stop temperature = 0.01, cooling factor = 0.99, spins = 17).^45^

In terms of the centrality of all depressive symptoms, the node strength centrality was defined as the sum of all associations of a given node with all other nodes. Additionally, the closeness centrality was defined as a measure of how close a symptom was to all other symptoms. The betweenness centrality was defined as the shortest length of a path connecting any two nodes. As the node strength centrality was a common and stable central metric and was substantially correlated with the closeness centrality or betweenness centrality, the most central symptoms within the network structures of the 10 depressive symptom profiles were estimated mainly based on node strength centrality. Centrality stability was operationally defined by the correlation stability coefficient (CS‐coefficient), as the CS‐coefficient denoted the maximum proportion of cases that could be eliminated to obtain a 95% probability that the ranking correlation between the original network and the case‐subset network would amount to a very large effect (0.7).^46^ Epskamp *et al*.^47^ recommended only interpreting centrality indices with a CS‐coefficient above 0.25, but preferentially above 0.5. Using 95% nonparametric bootstrap confidence intervals (1000 bootstrap samples) of the difference between each pair of centrality indices, significant differences among centrality indices were calculated.

### Ethics

All the institutional review boards of the Psychiatric Center, Taipei City Hospital, Taipei, Taiwan (receipt number: TCHIRB‐1020206‐E) and other participating survey centers approved the study protocol and informed consent forms. All participants signed the written informed consent forms before participation in this survey.

## Results

The 1174 Asian patients with depressive disorders included in the present study consisted of 240 Chinese, 38 Hong Kongese, 142 Japanese, 173 Korean, 38 Singaporean, 130 Indian, 111 Malaysian, 145 Thai, 50 Taiwanese, and 107 Indonesian individuals. Thus, according to the UN classification, the numbers of Asians overall and East Asians and South or Southeast Asians were 1174 (100%), 643 (54.8%), and 531 (45.2%), respectively. According to the World Bank income designation, Asian patients from high‐ and middle‐income countries were 441 (37.6%) and 773 (62.4%), respectively. The mean age of the participants was 48.3 (SD = 16.9) years. More than half of the participants were female (*n* = 696, 59.3%). A diagnosis of depressive episode (F32) was made in three‐quarters (*n* = 881, 75.0%), whereas a diagnosis of recurrent depressive disorder (F33) was made in one‐quarter (*n* = 293, 25.0%) of the cohort. In terms of treatment setting, the proportions of public and private settings were 74.5% (*n* = 875) and 25.5% (*n* = 299), respectively. In terms of hospital settings, the proportions of psychiatric, general, university‐affiliated psychiatric, and university‐affiliated general hospitals were 37.5% (*n* = 440), 10.8% (*n* = 127), 6.7% (*n* = 79), and 45.0% (*n* = 528), respectively. The abbreviations and the presence rates of the depressive symptom profiles are reported in Table 2.

**Table 2:** Rate of each of the ICD‐10 depressive symptom profiles based on geographic and economic classifications of Asian countries, *n* (%)

Depressive symptoms	Abbreviations	Asians overall (*n* = 1174)	Geographic classification		Economic classification	
			East (*n* = 643)	South or Southeast (*n* = 531)	HIC (*n* = 441)	MIC (*n* = 733)
Persistent sadness or low mood	SAD	859 (73.2)	484 (75.3)	375 (70.6)	341 (77.3)	518 (70.7)
Loss of interest or pleasure	INT	623 (53.1)	353 (54.9)	270 (50.8)	209 (47.4)	414 (56.5)
Fatigue or low energy	FAT	536 (45.7)	310 (48.2)	226 (42.6)	206 (46.7)	330 (45.0)
Disturbed sleep	SLE	748 (63.7)	406 (63.1)	342 (64.4)	265 (60.1)	483 (65.9)
Poor concentration or indecisiveness	CON	348 (29.6)	143 (22.2)	205 (38.6)	89 (20.2)	259 (35.3)
Low self‐confidence	SEL	268 (22.8)	157 (24.4)	111 (20.9)	98 (22.2)	170 (23.2)
Poor or increased appetite	APE	384 (32.7)	215 (33.4)	169 (31.8)	120 (27.2)	264 (36.0)
Suicidal thoughts or acts	SUI	268 (22.8)	158 (24.6)	110 (20.7)	87 (19.7)	181 (24.7)
Agitation or slowing of movements	AGI	267 (22.7)	160 (24.9)	107 (20.2)	141 (32.0)	126 (17.2)
Guilt or self‐blame	GUI	185 (15.8)	122 (19.0)	63 (11.9)	61 (13.8)	124 (16.9)

HIC, high‐income country; MIC, middle‐income country.

### Estimating a network of depressive symptom profiles in Asian patients with depressive disorder overall

**Figure 1:**
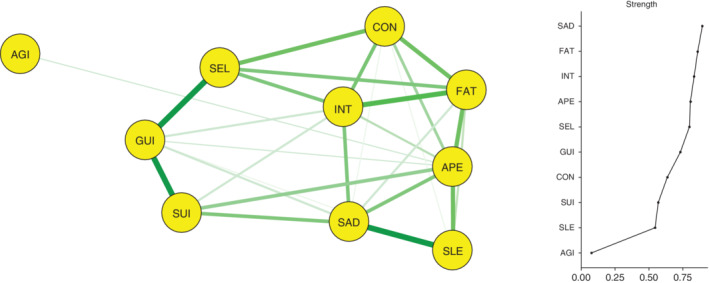
Network analysis of the depressive symptom profiles in Asian patients with depressive disorder overall (*n* = 1174). AGI, agitated or slowed movements; APE, decreased or increased appetite; CON, poor concentration or indecisiveness; FAT, fatigue or low energy; GUI, guilt or self‐blame; INT, loss of interest or pleasure; SAD, persistent sadness or low mood; SEL, low self‐confidence; SLE, disturbed sleep; SUI, suicidal thoughts or acts.

As shown in Figure 1, a psychopathological network consisting of the 10 depressive symptom profiles listed in the ICD‐10 diagnostic criteria for depression was constructed in 1174 Asian patients with depressive disorders, and 29 (64.4%) of the possible 45 edges were estimated to be above zero. The three strongest associations within the network were between INT and SUI, SEL and AGI, and FAT and GUI. A community‐detection analysis estimated that the 10 depressive symptoms were organized into two clinically meaningful clusters. With AGI being largely isolated, the largest cluster included the other nine depressive symptoms.

Node strength centralities of the 10 depressive symptoms are shown in Figure 1. SAD had the greatest node strength centrality in the network and was considered the most important symptom within the network. Following SAD, FAT and INT were most significantly centrally situated. In contrast, due to its virtual disconnection within the network, AGI had the lowest node strength centrality. The betweenness and closeness centralities for the 10 depressive symptom profiles are visualized in Figure S1. An excellent level of stability was reported for node strength centrality (i.e., CS‐coefficient = 0.594), although both betweenness (i.e., CS‐coefficient = 0.050) and closeness (i.e., CS‐coefficient = 0.050) centralities demonstrated low levels of stability.

### Estimating a network of depressive symptom profiles based on the geographic classification of Asian countries

**Figure 2:**
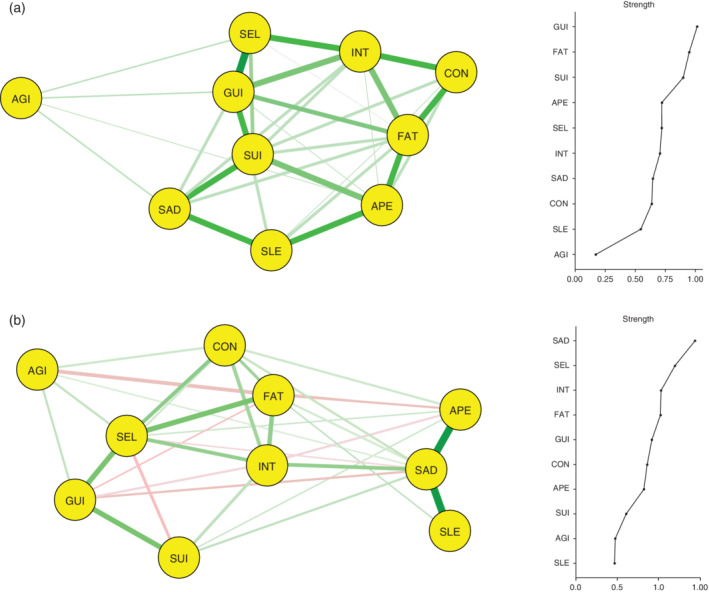
Network structure and node strength centrality of depressive symptom profiles based on the geographic classification of Asian countries: (a) East Asian patients with depressive disorder (*n* = 643) and (b) South or Southeast Asian patients with depressive disorder (*n* = 531). AGI, agitated or slowed movements; APE, decreased or increased appetite; CON, poor concentration or indecisiveness; FAT, fatigue or low energy; GUI, guilt or self‐blame; INT, loss of interest or pleasure; SAD, persistent sadness or low mood; SEL, low self‐confidence; SLE, disturbed sleep; SUI, suicidal thoughts or acts.

As shown in Figure 2a, a network consisting of the 10 depressive symptom profiles was constructed in 643 East Asian patients with depressive disorders, and 34 (75.6%) of the possible 45 edges were estimated to be above zero. The three strong associations were revealed between SEL and GUI, SUI and GUI, and FAT and APE within the network. A community‐detection analysis estimated a single clinically meaningful cluster consisting of all 10 depressive symptom profiles.

As shown in Figure 2a, by inspecting the node strength centralities of the 10 depressive symptom profiles, GUI, FAT, and SUI were reported as the top three central symptoms within the network. In contrast, AGI was the least centrally situated symptom within the network. The betweenness and closeness centralities for the 10 depressive symptom profiles are shown in Figure S2. An interpretable level of stability was reported by node strength centrality (i.e., CS‐coefficient = 0.360), although low levels of stability were reported for both betweenness (i.e., CS‐coefficient = 0.049) and closeness (i.e., CS‐coefficient = 0.128) centralities.

As shown in Figure 2b, a network consisting of the 10 depressive symptom profiles was constructed in 531 South or Southeast Asian patients with depressive disorders; 33 (73.3%) of the possible 45 edges were estimated to be above zero. The three strongest associations were noted between SAD and APE, SAD and SEL, and SEL and GUI within the network. A community‐detection analysis estimated that the 10 depressive symptoms were organized into three clinically meaningful clusters. Cluster A included five symptoms: INT, FAT, CON, SEL, and AGI. Cluster B included another three symptoms: SAD, SLE, and APE. Cluster C included two symptoms: SUI and GUI. As shown in Figure 2b, by inspecting the node strength centralities of the 10 depressive symptom profiles, SAD, SEL, and INT were reported as the top three central symptoms within the network. In contrast, SLE was the least centrally situated within the network. The betweenness and closeness centralities for the 10 depressive symptom profiles are depicted in Figure S3. However, an interpretable level of stability was reported by the node strength (i.e., CS‐coefficient = 0.345), although low levels of stability were reported for both betweenness (i.e., CS‐coefficient < 0.0001) and closeness (i.e., CS‐coefficient = 0.087) centralities.

### Estimating a network of depressive symptom profiles based on the economic classification of Asian countries

**Figure 3:**
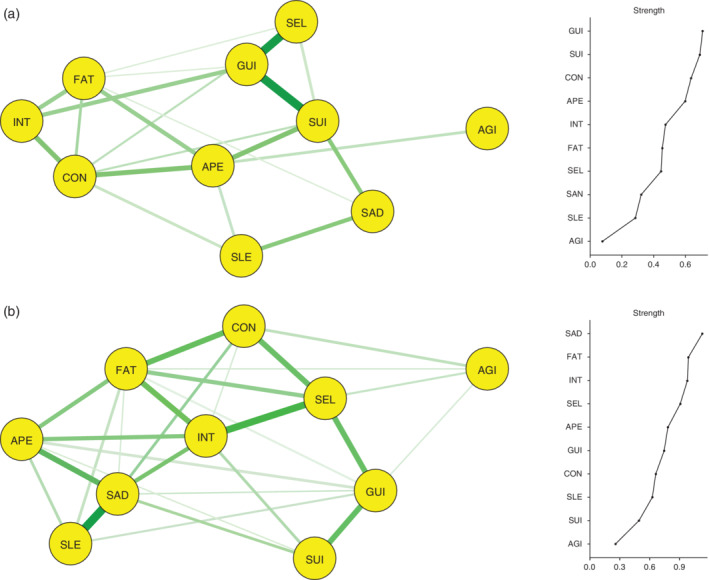
Network structure and node strength centrality of depressive symptom profiles based on the economic classification of Asian countries: Asian patients from (a) high‐income countries (*n* = 441) and (b) middle‐income countries (*n* = 733). AGI, agitated or slowed movements; APE, decreased or increased appetite; CON, poor concentration or indecisiveness; FAT, fatigue or low energy; GUI, guilt or self‐blame; INT, loss of interest or pleasure; SAD, persistent sadness or low mood; SEL, low self‐confidence; SLE, disturbed sleep; SUI, suicidal thoughts or acts.

As shown in Figure 3a, a network consisting of the 10 depressive symptom profiles was constructed in 441 high‐income‐country Asian patients with depressive disorders, and 22 (48.9%) of the possible 45 edges were estimated to be above zero. The three strongest associations were found between SEL and GUI, SUI and GUI, and INT and CON within the network. A community‐detection analysis estimated that the 10 depressive symptoms were organized into three clinically meaningful clusters. Cluster A included five symptoms: INT, FAT, CON, APE, and AGI. Cluster B included another three symptoms: SEL, SUI, and GUI. Cluster C included one symptom: SAD. As shown in Figure 3a, by inspecting the node strength centralities of the 10 depressive symptom profiles, GUI, SUI, and CON were reported as the top three central symptoms within the network. In contrast, AGI was the least centrally situated symptom within the network. The betweenness and closeness centralities for the 10 depressive symptom profiles are shown in Figure S4. An interpretable level of stability was reported by the node strength centrality (i.e., CS‐coefficient = 0.283), although low levels of stability were reported for both betweenness (i.e., CS‐coefficient = 0.127) and closeness (i.e., CS‐coefficient = 0.128) centralities.

As shown in Figure 3b, a network consisting of the 10 depressive symptom profiles was constructed in 773 middle‐income‐country Asian patients with depressive disorders; 29 (64.4%) of the possible 45 edges were estimated to be above zero. The three strongest associations were noted between SAD and SLE, INT and SEL, and SAD and APE within the network. A community‐detection analysis estimated that the 10 depressive symptoms were organized into one clinically meaningful cluster including all symptoms. As shown in Figure 3b, by inspecting the node strength centralities of the 10 depressive symptom profiles, SAD, FAT, and INT were reported as the top three central symptoms within the network. In contrast, AGI was the least centrally situated within the network. The betweenness and closeness centralities for the 10 depressive symptom profiles are depicted in Figure S5. However, interpretable levels of stability were reported for the node strength (i.e., CS‐coefficient = 0.283) and closeness (i.e., CS‐coefficient = 0.283) centralities, although a low level of stability was reported for the betweenness centrality (i.e., CS‐coefficient = 0.050).

## Discussion

In summary, in Asian patients with depressive disorders overall, SAD, FAT, and INT were among the top three central symptoms within the network of ICD‐10 depressive symptoms. Although these top‐three central symptoms of Asian patients with depressive disorders overall were equal to the three typical symptoms of the ICD‐10 diagnostic criteria for depressive episode,^8^ the top three central symptoms varied within each of the networks of depressive symptoms in East, South, or Southeast Asian patients with depressive disorders. In East Asian patients, GUI, FAT, and SUI were the top three central symptoms within the network. In South Asian patients, SAD, SLE, and AGI were the top three central symptoms within the network. In Southeast Asian patients, SAD, SEL, and CON were the top three central symptoms within the network.

### Typical symptoms of the diagnostic criteria for depressive episode as the most central symptoms within the network of depressive symptoms

The ICD‐10 diagnostic criteria for depressive episodes were supported by the network of depressive symptoms in Asian patients with depressive disorders overall (*n* = 1174), as indicated by an excellent level of node strength centrality stability. First, all the typical symptoms of the ICD‐10 diagnostic criteria for depressive episodes were the most centrally situated within the network structure of depressive symptoms. Second, within the network of depressive symptom profiles, loss of interest/pleasure and fatigue/loss of energy of the typical symptoms contributed to forming strong associations with other depressive symptoms. Third, a community‐detection analysis revealed that, excluding psychomotor disturbances, a depressive symptom constellation consisted of all other nine depressive symptoms. These findings partly supported the actual existence of the ICD‐10 diagnostic criteria for depressive episode as an interrelated symptom organization. Thus, our findings can support the provisional ICD‐11 diagnostic criteria for single‐episode depressive disorder,^48^ which is characterized by the depressive symptom profiles that are similar to the ICD‐10 diagnostic criteria.

However, as mentioned in the Introduction, it was repeatedly reported that there were no significant differences in node strength centralities between the DSM symptoms and non‐DSM symptoms within the network of depressive symptoms in patients with depressive disorders.^29^, ^30^ Since the network was estimated only using the ICD‐10 diagnostic criteria for a depressive episode, the differences in node strength centralities in our study cannot be discussed. Despite the differences in study samples, our findings were partly consistent with the findings of Garabiles *et al*.,^49^ in that fatigue was the most centrally situated within the network of the Patient Health Questionnaire‐9 items in migrant Filipino domestic workers.

### Geographic variations in networks of depressive symptoms

The networks of depressive symptoms were estimated differently with respect to geographic variations. Thus, we speculate that geographic variations in the networks of depressive symptoms could be associated with cultural influences on depressive symptoms. Although the heterogeneity of the culture and ethnicity diluted the effect of geographic differences in Asian countries,^50^, ^51^, ^52^, ^53^, ^54^ differences in network structures might be mostly attributed to the pathoplastic effects that denote the cultural contribution towards the modeling or plastering of manifestations of the depressive symptomatology.^33^

In East Asian patients with depressive disorders, GUI, FAT, and SUI were the most centrally situated within the network of depressive symptom profiles. However, SAD was not centrally situated within the network. These findings can be influenced by the following cultural factors: First, Chinese women constituted the most substantial portion of East Asians among our study participants. Contrary to the higher suicidal rate in men as compared to women globally,^55^ the reverse pattern has been reported among women in rural areas in Mainland China. Namely, a preponderance of women who completed suicides is a phenomenon limited to China.^56^ Second, as compared to American outpatients with MDD, Korean outpatients with MDD were characterized by more prevalent suicidality and hypochondriasis, and less prevalent depressed mood.^57^ Third, in terms of the ‘intersection of a collectivistic society encountering an individualistic performance‐based system,’ modern‐type depression (MTD) has been concurrently proposed as a culture‐specific phenomenon that is prevalent in the younger Japanese generation. MTD is characterized by mild to moderate depressive episodes combined with fatigue, blaming others, impulsive suicidal actions, and so forth.^58^, ^59^ Importantly, religious affiliations have been proposed as the most important cultural factor for affecting human experience, behavior, and illness patterns.^60^ Although religious affiliations and other cultural contexts have been influenced by colonization, globalization, and industrialization,^33^ from the viewpoint of the Freudian theory, it has been suggested that the structure of neurotic depression can be continually affected by Confucianism under the influences of the Sinosphere in East Asians.^61^ Moreover, it is known that Confucianism has the paradoxical effects of reducing suicidal ideations and increasing the stigma for suicidal survivors.^62^ Thus, it is speculated that a unique depressive symptom constellation, centrally involving guilt, loss of interest, and suicidality, is present in East Asian patients with depressive disorders. Under the influence of Confucianism or Neo‐Confucianism, it is the ‘face’ rather than the ‘mood’ that matters most, resulting in the suppression of depressive symptoms. The ‘loss of face’ is a sign of ‘remorse’ or ‘guilt’ and is sometimes accompanied by ‘suicidal ideas’ in Koreans.^57^ Similarly, ‘neurasthenia’ or the fatigue syndrome in the Chinese^63^ is also strongly related to depression. Such are the unique cultural expressions of distress and depression in this region.

In South or Southeast Asian patients with depressive disorders, SAD, SEL, and INT were the most centrally situated within the network of depressive symptom profiles. In the South or Southeast Asian region, the heterogeneity of the ethnicity and/or culture has been remarkable. For example, despite being one country, India has more than 100 different ethnicities and more than eight religions, and Malaysia has more than three ethnicities and more than five different religions.^64^ Thus, although these findings cannot be simply explained, they may be partly based on the report that depressive symptom profiles of South Asians were characterized by significant preponderances of loss of interest, poor concentration, and poor appetite compared to those of East and Southeast Asians in other findings of the REAP‐AD survey.^64^ In 488 Indian elderly patients with depression, ‘feeling tired or having little energy’ was the most prevalent depressive symptom, followed by ‘not being able to stop or control worry,’ ‘trouble sleeping,’ ‘trouble relaxing,’ ‘worry too much about different things,’ and others.^65^ Also, these findings suggest that rather than fatigue or loss of energy, persistent sadness or low mood and loss of interests or pleasure are the most central symptoms among the ICD‐10 diagnostic criteria for depressive episodes for South or Southeast Asian patients with depressive disorders. In terms of a strong association between depressed mood and MDD, low socioeconomic status and social isolation have been proposed as risk factors for depressed mood.^66^ Thus, our findings suggest that approaches that address depressed‐mood‐associated clinical factors may be helpful for evaluating and treating South or Southeast Asian patients with depressive disorders. Moreover, bleak and pessimistic views in the ICD‐10 criteria may be consistent with hopelessness, which has newly been added as a subjective descriptor for depressed moods in the DSM‐5 criteria. Thus, despite the heterogeneity of the ethnicity and culture, the network structure of South or Southeast Asian patients with depressive disorders may support the DSM‐5 diagnostic criteria for MDD.

### Economic variations in networks of depressive symptoms

Differences in geographic and economic classifications of Asian countries/SAR were only based on the trade‐off between China and Singapore in our study. Thus, the network structure of depressive symptoms in East Asian patients with depressive disorders has been similar to that in Asian patients from high‐income countries, whereas the network structure in South or Southeast Asian patients has been similar to that in Asian patients of middle‐income countries. Thus, as mentioned earlier, the network structure of depressive symptom profiles in Asian patients of high‐income countries may be partly supported by clinical characteristics of Japanese or Korean patients with depressive disorders^57^, ^58^, ^59^ and influenced by Confucianism or Neo‐Confucianism.^61^, ^62^ Furthermore, the network structure in Asian patients of middle‐income countries may be partly supported by clinical characteristics of Chinese, Indian, or Malaysian patients with depressive disorders.^56^, ^64^, ^65^ Although completed suicides have been predominant in Chinese women, suicidal ideation has been considered to be an independent factor of depression outside of high‐income countries.^66^ Thus, based on this relationship between suicidal ideation and depression outside of high‐income countries, it is speculated that SUI has been the second least central domain within the network structure of depressive symptom profiles.

### Limitations

Our study had several limitations as follows: First, the estimated network structures can change within the clinical course of depressive disorders. Reanalysis of the STAR*D clinical trial has reported that, whereas energy‐related symptoms are the most centrally situated at baseline, mood‐related symptoms are the most centrally situated at the end‐point.^67^ However, in our study, depressive disorder patients with any clinical course were included as study participants. Second, some of the ICD‐10 depressive symptoms have been aggregated (e.g., combining decreased and increased appetite). However, the depression‐related increase and decrease in appetite has been differentiated in terms of the patterns of aberrant activity in reward and interoceptive neurocircuitry.^68^ Thus, aggregated depressive symptoms should be separated in further network analyses of depressive symptoms. Third, information for the outdegree centrality and indegree centrality cannot be provided by the estimated centralities. Fourth, since a low level of stability has been reported for the node strength centrality (i.e., CS‐coefficient = 0.046) in a network of depressive symptom profiles in South Asian patients with depressive disorders, a network has been estimated in South or Southeast Asian patients with depressive disorders. Stability of the node strength centrality is defined not by a proper sample size but by a criterion of CS‐coefficient > 0.25,^47^ whereas the sample size (*n* = 130) of South Asian patients with depressive disorders was the smallest. Thus, the possibility that the heterogeneity of the ethnicity and culture may influence the network structure of depressive symptom profiles is not excluded. Fifth, although the REAP‐AD survey has recruited the study participants with a convenient sampling method, a potentiality of sampling bias cannot be excluded.

### Conclusions

Despite these limitations, our study shows that the estimated network of depressive symptoms among Asian patients with depressive disorders supports the ICD‐10 diagnostic criteria for depressive episode. In addition, the provisionally defined ICD‐11 diagnostic criteria for a single‐episode depressive disorder are supported by this study's findings. However, geographic variations in the networks of the depressive symptoms were present. Herein, although the heterogeneity of the ethnicity and culture may contribute to dilutions of geographic differences in clinical manifestations of depressive disorders, cultural contexts, including religious affiliations, can contribute to differences in network structures of depressive symptoms among Asian patients with depressive disorders. Most importantly, it is speculated that a unique depressive symptom constellation may influence the network structure of depressive symptom profiles, which is partly inconsistent with the ICD‐10 or DSM‐5 criteria, in East Asian patients or high‐income‐country patients with depressive disorders.

## Disclosure statement

The authors have no conflicts of interest to declare.

## Author contributions

S‐C.P., Y‐T.X., S.K., M‐Y.C., S‐K.L., S‐Y.Y., A.A., S.G., R.A.K., P.U., K.Y.C., A.J.T., C‐H.T., K.S., N.S., Y.C.P., and N.S. designed and conducted the study. S‐C.P., E.Y.J., and Y.C.P. performed analysis. S‐C.P., E.Y.J., and Y.C.P. wrote the manuscript. All authors read and approved the final manuscript.

## Supporting information


**Figure S1.** Node strength, betweenness, and closeness centralities of the 10 depressive symptom profiles in Asian patients with depressive disorder overall (*n* = 1174).
**Figure S2.** Node strength, betweenness, and closeness centralities of the 10 depressive symptom profiles in East Asian patients with depressive disorder (*n* = 643).
**Figure S3.** Node strength, betweenness, and closeness centralities of the 10 depressive symptom profiles in South or Southeast Asian patients with depressive disorder (*n* = 531).
**Figure S4.** Node strength, betweenness, and closeness centralities of the 10 depressive symptom profiles in Asian patients from high‐income countries (*n* = 441).
**Figure S5.** Node strength, betweenness, and closeness centralities of the 10 depressive symptom profiles in Asian patients from low‐income countries (*n* = 733).
