# Supplementary material for: Network analysis of the depressive symptom profiles in Asian patients with depressive disorders: Findings from the Research on Asian Psychotropic Prescription Patterns for Antidepressants (REAP‐AD)
Source: Psychiatry Clin Neurosci. 2020 Mar 5;74(6):344–53. doi: 10.1111/pcn.12989 (PMC7318233; doi:10.1111/pcn.12989)
Supplement: Supplementary file 1 — Figure S1. Node strength, betweenness, and closeness centralities of the 10 depressive symptom profiles in Asian patients with depressive disorder overall (n = 1174). Figure S2. Node strength, betweenness, and closeness centralities of the 10 depressive symptom profiles in East Asian patients with depressive disorder (n = 643). Figure S3. Node strength, betweenness, and closeness centralities of the 10 depressive symptom profiles in South or Southeast Asian patients with depressive disorder (n = 531). Figure S4. Node strength, betweenness, and closeness centralities of the 10 depressive symptom profiles in Asian patients from high‐income countries (n = 441). Figure S5. Node strength, betweenness, and closeness centralities of the 10 depressive symptom profiles in Asian patients from low‐income countries (n = 733). [file PCN-74-344-s001.docx]

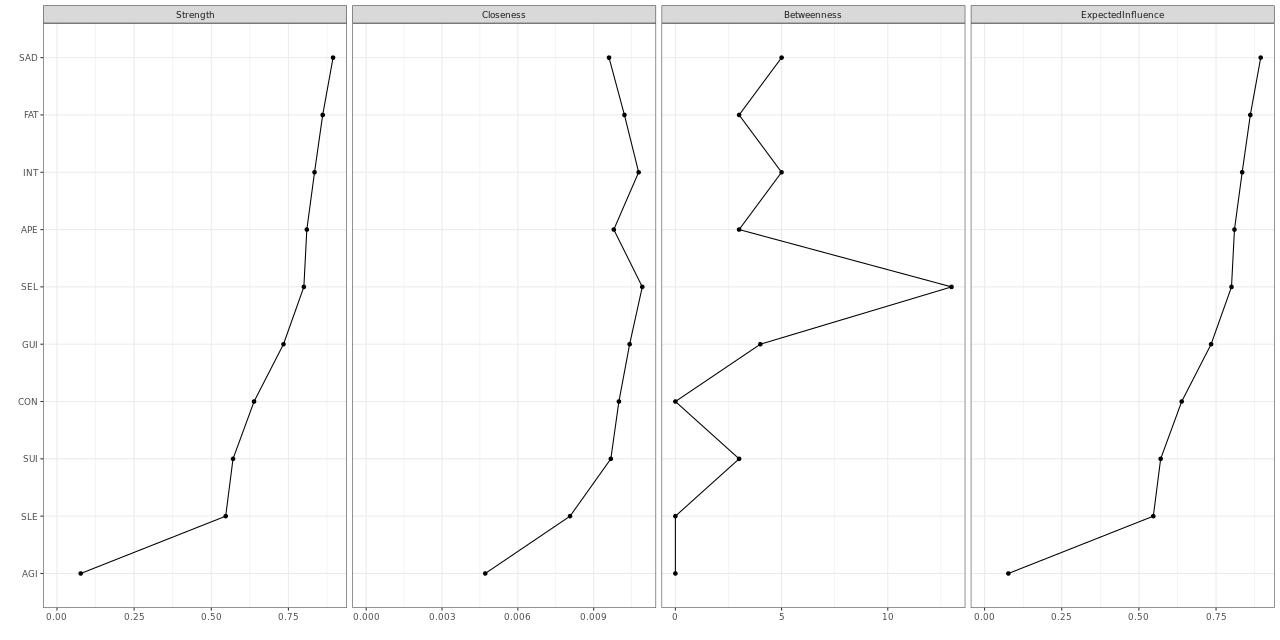


**Fig. S1.** Node strength, betweenness, and closeness centralities of the ten depressive symptom profiles in Asian patients with depressive disorder overall (n = 1,174)

***Abbreviations;* AGI**, agitation or slowing of movements; **APE**, poor or increased appetite; **CON**, poor concentration or indecisiveness; **FAT**, fatigue or low energy; **GUI**, guilt or self-blame; **INT**, loss of interests or pleasure; **SAD**, persistent sadness or low mood; **SEL**, low self-confidence; **SLE**, disturbed sleep; **SUI**, suicidal thoughts or acts


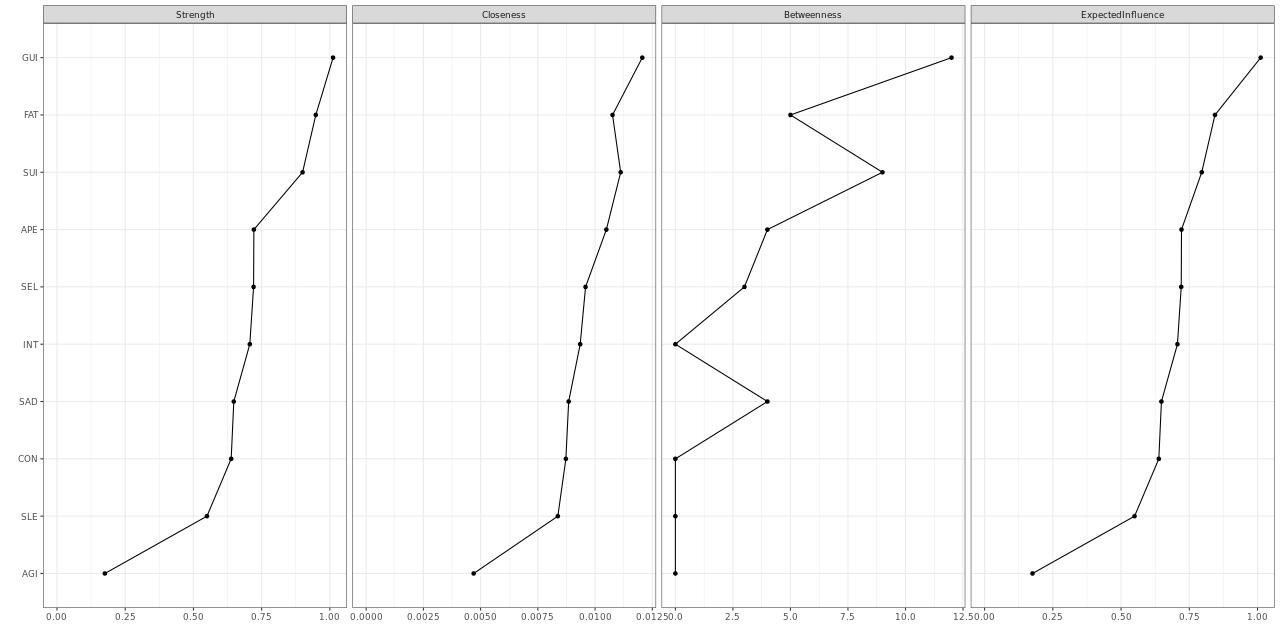


**Fig. S2.** Node strength, betweenness, and closeness centralities of the ten depressive symptom profiles in East Asian patients with depressive disorder (n = 643)

***Abbreviations;* AGI**, agitation or slowing of movements; **APE**, poor or increased appetite; **CON**, poor concentration or indecisiveness; **FAT**, fatigue or low energy; **GUI**, guilt or self-blame; **INT**, loss of interests or pleasure; **SAD**, persistent sadness or low mood; **SEL**, low self-confidence; **SLE**, disturbed sleep; **SUI**, suicidal thoughts or acts


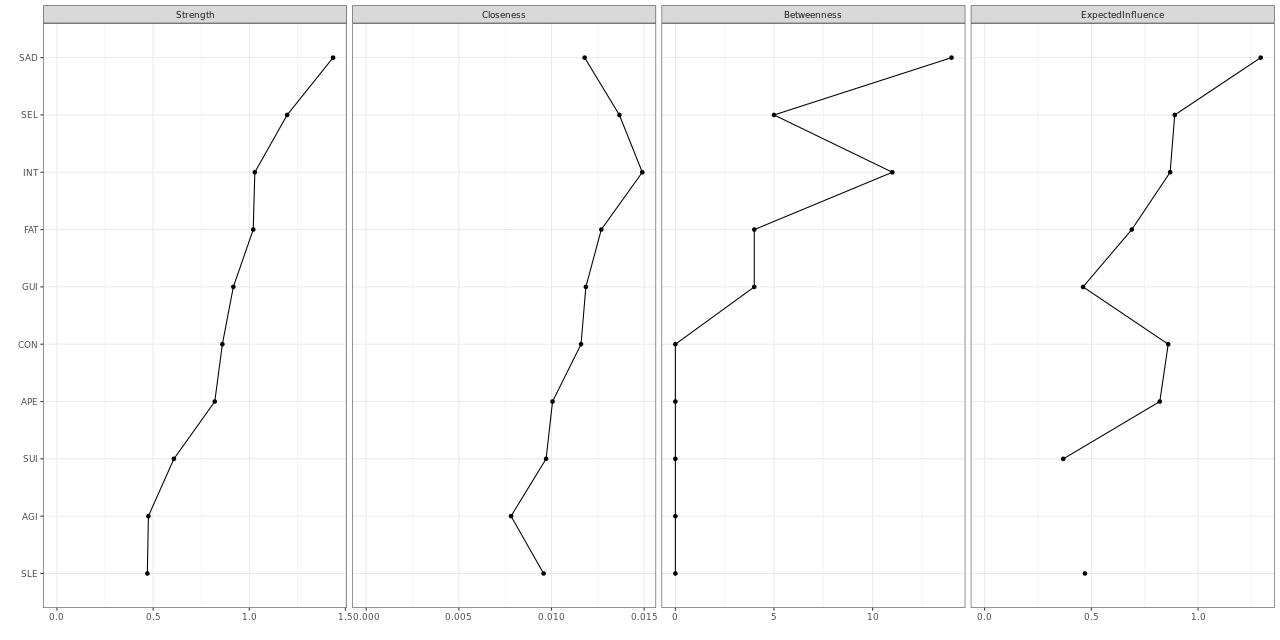


**Fig. S3.** Node strength, betweenness, and closeness centralities of the ten depressive symptom profiles in South or Southeast Asian patients with depressive disorder (n = 531)

***Abbreviations;* AGI**, agitation or slowing of movements; **APE**, poor or increased appetite; **CON**, poor concentration or indecisiveness; **FAT**, fatigue or low energy; **GUI**, guilt or self-blame; **INT**, loss of interests or pleasure; **SAD**, persistent sadness or low mood; **SEL**, low self-confidence; **SLE**, disturbed sleep; **SUI**, suicidal thoughts or acts


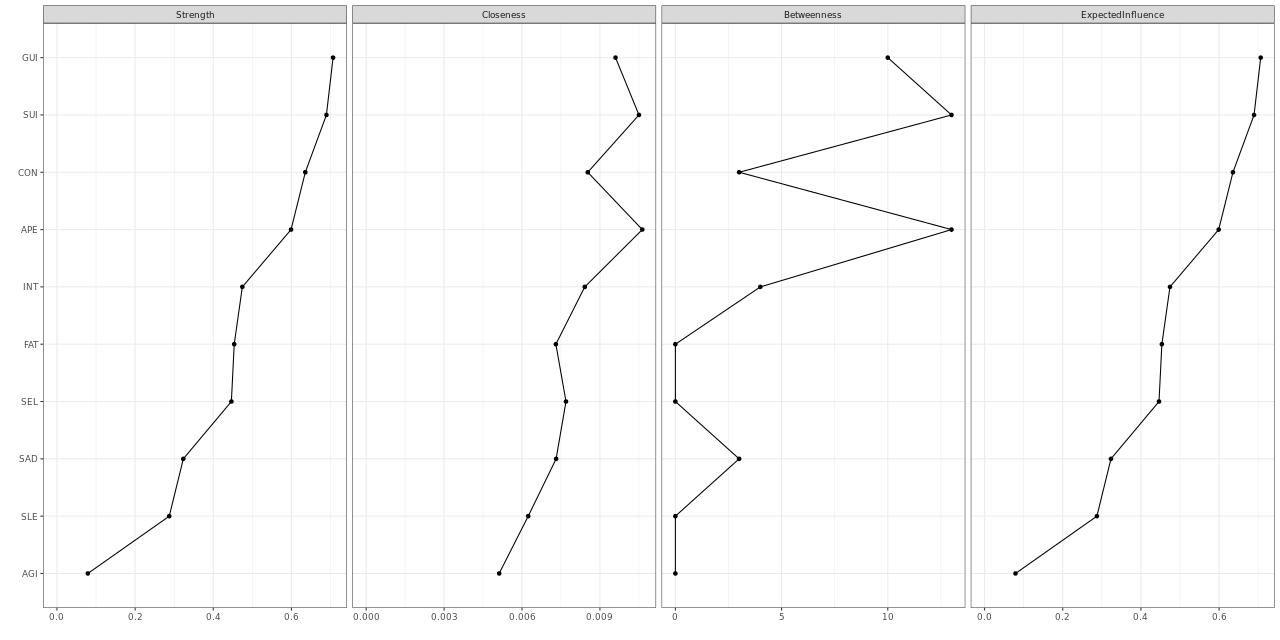


**Fig. S4.** Node strength, betweenness, and closeness centralities of the ten depressive symptom profiles in Asian patients from high-income countries

***Abbreviations;* AGI**, agitation or slowing of movements; **APE**, poor or increased appetite; **CON**, poor concentration or indecisiveness; **FAT**, fatigue or low energy; **GUI**, guilt or self-blame; **INT**, loss of interests or pleasure; **SAD**, persistent sadness or low mood; **SEL**, low self-confidence; **SLE**, disturbed sleep; **SUI**, suicidal thoughts or acts


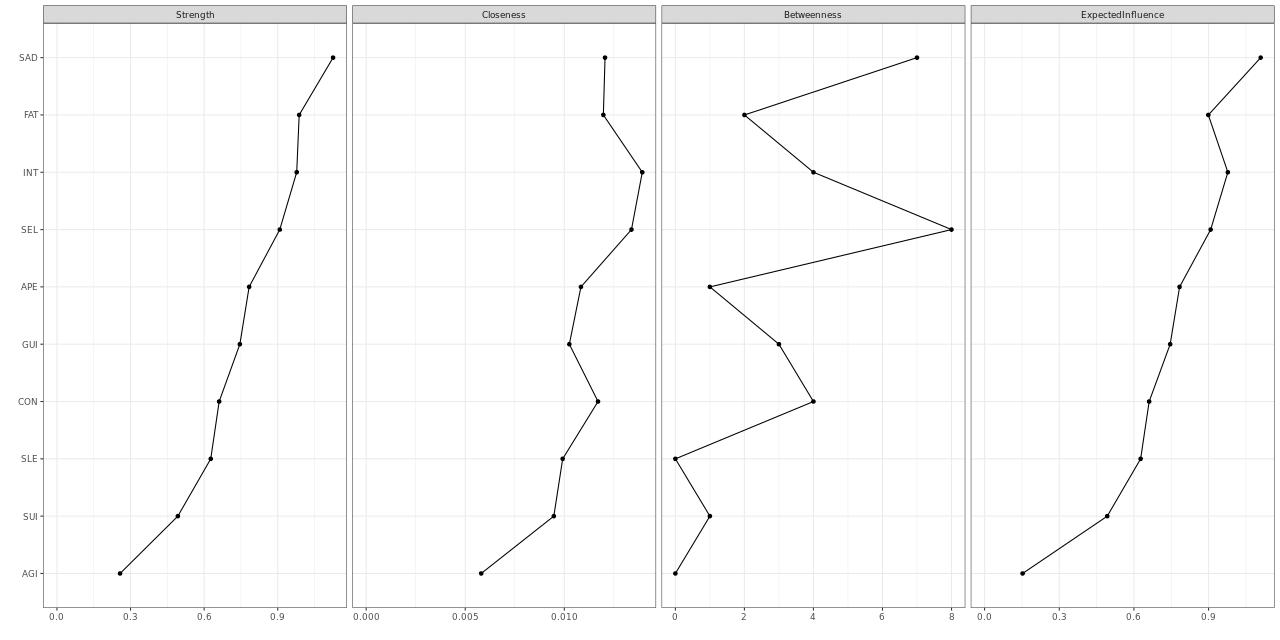


**Fig. S5.** Node strength, betweenness, and closeness centralities of the ten depressive symptom profiles in Asian patients from low-income countries

***Abbreviations;* AGI**, agitation or slowing of movements; **APE**, poor or increased appetite; **CON**, poor concentration or indecisiveness; **FAT**, fatigue or low energy; **GUI**, guilt or self-blame; **INT**, loss of interests or pleasure; **SAD**, persistent sadness or low mood; **SEL**, low self-confidence; **SLE**, disturbed sleep; **SUI**, suicidal thoughts or acts
